# St. Bartholomew's Hospital

**Published:** 1898-11-12

**Authors:** 


### ST. BARTHOLOMEW'S HOSPITAL.

### STRUCTURAL IMPROVEMENTS IN THE SOUTH WING.

Very fresh and bright is the appearance of the south wing at St. Bartholomew's Hospital since the paraphernalia of the builder and painter have been removed. In some places, as in the sisters' rooms, the wooden partitions have been replaced by brickwork, which is cemented and painted, and in the lift iron trellis-work gates have been placed. The colouring throughout the wards and staircases is a light soft shade of green for the upper portion of the walls with a darker dado of the same. The flooring also has been renewed within the last year or two, and is now becoming darker, an improvement from an artistic point of view. The Doulton stoves, which we described in our Christmas number last year, are working successfully and look very pretty, the open fire being combined with a fresh air inlet. The substitution of white enamel bedsteads fitted with a wire woven mattress of a more open mesh than usual is a distinct improvement. The beds made by this kind of mattress are very firm. The bedsteads are mounted on rollers, which are practically small wheels, instead of castors, and are consequently easier to move, and they do not injure the flooring. The new lockers are much liked. They have the appearance of a chair. The back is about four inches deep, and is divided, a couple of pigeon-holes opening on the side next the bed. On the top is a thick glass slab whereon the glasses, &c , may stand, and which is easy to keep clean. A flap-leaf of the same size as the chair back is so arranged that it can be raised and kept in position by a bracket, and thus used as a bed-table, whilst the seat itself lifts up, disclosing a receptacle for garments. The new heating coils were likewise described in our number of last Christmas. The ventilators open immediately behind them, and the inlet of air is regulated at pleasure; neither here nor elsewhere, however, is the air filtered. The walls of the operating theatre in this wing have been faced with a kind of alabaster of fine texture, and with the appearance of sienna marble, it looks well. The fire-place here is also new. It is an open grate without fixtures. The students' galleries are of iron, painted red, and not of recent date. There are a couple of operating tables, one is of metal and glass, whilst the dressing tables are all in those materials. There is no system of artificial ventilation in the theatres. New cooking ranges, with ovens and boilers attached, have been added to the ward kitchens. Special arrangements to guard against fire have been made, buckets and a "corridor fire pump" have been placed on the landings, and when complete, communication will be established with the Metropolitan Fire Brigade Station in Whitecross Street. It is possible that a permanent fireman may be appointed.

